# How resource limitations and household economics may compromise efforts to safeguard children during outbreaks

**DOI:** 10.1186/s12889-019-7968-6

**Published:** 2020-02-24

**Authors:** Kellen Myers, Agnesa Redere, Nina H. Fefferman

**Affiliations:** 1 Department of Ecology & Evolutionary Biology, University of Tennessee, Knoxville, TN USA; 2 Department of Mathematics, University of Tennessee, Knoxville, TN USA; 3 Department of Ecology, Evolution, and Natural Resources, Rutgers University, New Brunswick, NJ USA; 4 National Institute for Mathematical and Biological Synthesis, University of Tennessee, Knoxville, TN USA

**Keywords:** Epidemiology, Household economic effects, Resource management, Targeted treatment strategies

## Abstract

**Background:**

Epidemiological models have been employed with great success to explore the efficacy of alternative strategies at combating disease outbreaks. These models have often incorporated an understanding of age-based susceptibility and severity of outcome, considering how to limit the adverse outcomes or disease burden relative to an age structure. Such models frequently recommend the preferential treatment/vaccination of children or the elderly, demonstrating how prevention of serious disease within these etiological subgroups can provide both protection within the subgroup itself and indirect protection to the broader population. However, it is most frequently the case that these target populations are consumers, rather than providers, of household resources. In areas of the globe where continued health of household members relies on continued provision of resources, these models may fail to provide the most effective overall strategies for health outcomes in both target populations and overall. This is particularly important for tropical diseases impacting rural and low-income areas in which the disease may be endemic or newly emergent, particularly in the wake of natural disasters.

**Methods:**

We propose a modified epidemiological model with targeted treatment in resource-limited populations. We evaluate the model over a broad parameter space.

**Results:**

This model demonstrates how economic limitations may shift the optimal strategy. It may be advantageous to treat populations at lesser direct risk if they are responsible for providing secondary protection to higher-risk population(s) by producing household resources. Evaluation of this model over the parameter space reveals that, in some cases, targeting treatment towards consumers may result in greater numbers of consumer infections.

**Conclusions:**

Our results demonstrate how household resource limitation can drastically affect the impact of targeted treatment strategies for limiting epidemics. Depending on the economic circumstances, it is possible that focusing treatment on consumers such as children can produce a counter-intuitive outcome in which more children contract the disease.

## Background

Mathematical epidemiological models have a long history of examining the impact of both prophylaxis and treatment on the dynamics of disease within populations [1–5]. These examinations have incorporated certain aspects of financial concerns, usually when considering cost-benefit analyses of population-level investment into particular interventions, such as vaccination [6–8]. Many economic epidemiology models have been built to explore both targeted and non-target specific vaccination to determine maximal effectiveness at population-wide reduction of both incidence and/or disease-related mortality [9–11]. Similarly, models of treatment, especially under assumptions of limited resources, whether economic or pharmaceutical, have explored how best to reduce total impact of epidemic outbreaks for many different diseases and in many different populations [12–15].

Models have also demonstrated the different needs of different demographically distributed populations, especially in diseases with age- [16, 17] or immunocompentence-specific [18, 19] etiologies. The impact of differential treatment protocols has been studied before, demonstrating effects within and without the particular etiological group being treated [20, 21]. Nowhere are these differences more extreme than in developing or war-torn nations, where populations are frequently exposed to a broad variety of pathogens with serious outcomes. When these outcomes are so skewed as to risk the lives of children, while merely causing moderate, short-duration incapacity in adults, these models have frequently focused on limiting disease-related mortality [22–26], mirroring public sentiment and behavior (cf. [27–29]). While this is obviously one of the most important concerns, in severely socio-economically depressed areas, these strategies may prove economically naïve, leading to increased mortality from indirect negative health outcomes to children by compromising the health of their caretakers. Many of the areas of the world with severe socio-economic depression are also those facing a multitude of severe diseases. It is therefore important to couple epidemiological and economic models to investigate the dynamics as these two coupled systems impact economic productivity and health status of individuals in households.

Household economics and family health can be inextricably intertwined (cf. [30–33]): if the person responsible for the majority of a family’s income is unable to work due to disease, even if that disease provides no direct mortality risk to the infected individual, the loss of financial productivity can compromise, for example, the health and/or nutritional resources available to the family. In areas where minimal nutritional needs are difficult to meet under normal conditions, compromised nutritional intake can lead to increased disease susceptibility (cf. [34]). Models of endemic disease and economic distress at a national scale demonstrate that certain conditions create so-called “poverty traps” where disease and poverty are mutually reinforcing [35]. This leads to an obvious trade off in patterns of directed treatment, even if the only goal is the minimization of childhood disease burden (i.e. minimizing the greatest mortality risks). (Note: In the cases we examine here, we assume the disease affects the entire population rather than, for example, childhood diseases in which resource limitations do not relate to treatment allocation to specific demographic segments.)

From this perspective, population-level efforts to minimize the disease risk could require a shift from a purely epidemiological perspective to an economically motivated epidemiological perspective. Often, a health organization provides aid or assistance to supplement available resources to families. When such treatment resources are limited, the recommendation to focus treatment on those at greatest risk of direct disease-related mortality may inadvertently lead to greater overall mortality due to indirect economically-based effects on pathogen susceptibility such as those previously described. This is true even when strictly limited to only same-pathogen attributable mortality.

Models examining the impact of infection in different demographic segments of a single, interacting population, have shown that focusing prophylaxis or treatment on subpopulations in which there is heightened transmission of disease (through either increased contact or susceptibility) can have protective effects for the entire population (e.g. [20]). Since the most frequent subpopulation of concern for heightened transmission in these ways is children, there is the potential for conflict between family health and public health. If a family can do best at safeguarding all its members, including its children, by ensuring their economic safety by preferentially treating the parents, then that effect may spill out into the greater community. The potential for this interactive effect between household economics, household health, and community health implies that simple discount factors are insufficient to capture the true effects of household financial concerns on population-level outbreaks. A true epidemiological model, incorporating household income effects, is needed.

These models are inspired by a number of diseases for which resource management and treatment targeting is essential to the maintenance of a productive workforce. For example, malaria infection is less severe in able-bodied adults, but it will remove them from the workforce and hamper their household economic productivity. However, we do not study malaria specifically, instead focusing on a more general model. Diseases like leptospirosis and Chagas disease have been shown to be emergent and neglected in both Europe and the United States [36, 37], while malaria can be found to be seasonally emergent, rather than endemic, in parts of Africa and Asia [38, 39]. Treatment strategies for these diseases requires similar economic-epidemiological consideration. These effects are amplified by the increasing frequency, severity, and risk associated with natural disasters, like Hurricane Maria in Puerto Rico, which resulted in significant outbreaks of leptospirosis.

## Methods

Assuming that each member of a family has an economic role within the household as either a net producer of resources, or else as a net consumer, and taking into account not only direct costs of treatment for particular diseases, but also the costs of minimal nutritional maintenance, we propose a model that builds off of the standard SIR framework, splitting each disease state to separately characterize the producer and consumer populations within each household.

Compartmental models in epidemiology, whether implemented as continuous differential equations models, discrete agent based models, or otherwise, comprise a broad swathe of the literature in this area [40–43].

While our model is explicitly designed to consider preferential treatment strategies in response to the emergence of an infectious disease, the result is the same as models likewise tuned for preventative strategies for seasonal or ongoing endemic transmission by simply adjusting the economic parameters (e.g. subtracting preventative treatment costs from household income) and perhaps elongating the time frame over which we would estimate the disease burden.

### Discrete, agent-based simulation

We first consider a stochastic, discrete-time, agent-based model. 500 households are constructed randomly, with 1–4 producers and 0–7 consumers, drawn from a weighted binomial distribution, averaging 2 producers and 3 consumers per household. Households are generated randomly in this way to represent the probability that individuals “arrive” successfully in the household, with parameters adjusted to set the average and range accordingly. Such Bernoulli events produce a binomial distribution [44] and the details of this implementation can be found in Additional file 1. One producer in one household is initially infected (untreated) to begin the outbreak.

In order to understand the effect of age, particularly the division between ages associated with economic activity and inactivity, we include assumed age-based etiology for both producer and consumer populations (assuming producers are adult and consumers are children): probabilities of transmission *β*_*p*_ and *β*_*c*_, and of recovery *γ*_*p*_ and *γ*_*c*_. Infected individuals receiving treatment have increased probabilities of recovery ${\gamma}_p^{+}$ and ${\gamma}_c^{+}$. We also assign for each population a percentage of treatment coverage, *T*_*p*_ and *T*_*c*_, describing what portion of infected individuals from that population are intended to be treated. Individuals who are infected are categorized as treated with coverage *T*_*p*_ or *T*_*c*_ (respectively) at the time they become infected.

Further, we account for the interaction of household members, employing a different probability of transmission from infected to susceptible individuals, *ι* rather than *β*. We assume that household contact is frequent enough that individuals within a household are equally likely to transmit the infection to one another regardless of whether or not they are well enough to work. For this reason, we do not need to divide the population based on whether each producer works within the home or outside, which could vary from household to household, or even within a household.

With this framework, we then introduce the limitation of household economics, employing a total monetary resource per household (denoted *M*_*h*_(0) = 500), increased daily by healthy producers at rate *P*_*p*_ = 50, and decreased daily by costs to support consumers at rate *C*_*c*_, which will vary. We assume that infected producers do not produce any resources while sick. Further, we assume a fixed cost per treatment *C*_*t*_, and that treatment will only successfully be applied at initial onset of illness. To incorporate the indirect loss of immuno-support from lack of resources, we assumed an increased probability of transmission $\left\{\hat{\beta},\hat{\iota}\right\}$, to susceptible individuals in families without available resources. Increases in disease shedding and susceptibility (and thus transmission) are associated with lack of treatment and nutritional/economic stress [45, 46].

An underlying assumption of such a transmission process is that the population is relatively well-mixed, and that the social contact network between individuals is relatively uniform and well-connected. Because we are focusing on small populations, generally working in agricultural or other small, single-industry communities, we expect that there will be one school, one marketplace, one main workplace, etc. within the community, suggesting such a well-mixing assumption (a relatively common assumption in SIR and similar models) is particularly well-founded for this type of population.

At each time-step (1 day), we update the monetary resources of each household based on its producers, consumers, and treatment costs (if any). We vary the economic parameters *C*_*c*_ and *C*_*t*_ in our analysis. In addition to the economic dynamics, at each time-step, we associate to each individual a single probability derived from the transmission probabilities, and each individual randomly contracts the disease (or not) based on that composite probability. If so, the individual is randomly assigned a treatment status (treated or untreated) using the appropriate coverage proportion, *T*_*p*_ or *T*_*c*_. Parameter values and ranges of values are provided in Table 1. Note that these parameter values are used for both the discrete model and the continuous model introduced in the next subsection. Each rate is adjusted accordingly, translating the rates of a continuous model into the probabilities of a discrete model, so that the two models are equivalent (meaning the same basic reproduction ratio *R*_0_). To achieve this, we need only adjust the recovery rates (although one could also adjust transmission rates while preserving *R*_0_).

**Table 1:** Epidemiological and economic parameter values

Epidemiological Parameters		
% Producers in Population		40%
% Consumers in Population		60%
Transmission Probability (Producers)	*β*_*p*_	0.3
Transmission Probability (Consumers)	*β*_*c*_	0.5
Transmission Probability (Broke Producers)	${\hat{\beta}}_p$	0.4
Transmission Probability (Broke Consumers)	${\hat{\beta}}_c$	0.7
Recovery Probability (Producers)	*γ*_*p*_	0.1
Recovery Probability (Consumers)	*γ*_*c*_	0.1
Recovery Probability (Treated Producers)	${\gamma}_p^{+}$	0.5
Recovery Probability (Treated Consumers)	${\gamma}_c^{+}$	0.5
Transmission Probability (Household Producers)	*ι*_*p*_	0.03
Transmission Probability (Household Consumers)	*ι*_*c*_	0.05
Transmission Probability (Broke Household Producers)	${\hat{\iota}}_p$	0.04
Transmission Probability (Broke Household Consumers)	${\hat{\iota}}_c$	0.07
Treatment Proportion (Producers)	*T*_*p*_	*varies*
Treatment Proportion (Consumers)	*T*_*c*_	*varies*
Economic Parameters		
Producer Net Daily Production	*P*_*p*_	50
Consumer Net Daily Consumption	*C*_*c*_	*varies*
Cost of Treatment	*C*_*t*_	*varies*

Since our focal question relies on a careful balance between economic resources, costs of treatment, average household demographics, and the epidemiology of a treatable infection, we refrained from parameterizing the model in direct representation of any one outbreak. Instead, we have chosen to demonstrate the potential issues using assumed, abstracted figures that could easily approximate a variety of infectious disease outbreaks in different populations, but make particular predictions about no one specific epidemic.

In order to assess the effectiveness of treatment strategies, we fix a particular combination of *C*_*c*_ and *C*_*t*_ and run a set of Monte Carlo simulations across the full set of possible *T*_*p*_ − *T*_*c*_ combinations. We can observe the total number of infected individuals, or isolate the consumer population. We focus on the latter, since it is our objective to reduce infections in this more vulnerable population.

In order to produce meaningful data for a single *C*_*c*_ − *C*_*t*_ pair, where the Monte Carlo data begins to converge and show meaningful trends, we must run about 500 Monte Carlo simulations per *T*_*p*_ − *T*_*c*_ combinations, or roughly 200,000 simulations. The limitations of such high-intensity computation require us to modify our approach to analyze a greater portion of the space of *C*_*c*_ − *C*_*t*_ combinations.

Code used to produce this simulation is available in Additional file 1.

### Continuous approximation of the system

In order to more fully explore the range of impacts on such a system of varying the parameters governing the household economic constraints and targeted public health policies, we also constructed a continuous model of this system using ordinary differential equations. This continuous model complements the discrete model by providing greater analytic insight, while the discrete, agent-based model allows us to verify that our observations are present in a treatment of the system allowing for complete stochasticity and heterogeneity.

We denote the sizes of susceptible, infective, and recovered populations of producers and consumers as {*S*_*p*_, *I*_*p*_, *R*_*p*,_*S*_*c*_, *I*_*c*_, *R*_*c*_}, respectively. We designate infected individuals who are receiving treatment with a ^+^. We also denote the average household size ∣*h*∣ (which is 5, as above) and use this to define {*S*_*p*_, *I*_*p*_, *R*_*p*,_*S*_*c*_, *I*_*c*_, *R*_*c*_}, an average measure of the distribution of each class of individual in each household, scaled to the size of the total population, *N* = 2500. (Note: whereas the agent-based model allowed us to fully explore heterogeneity among households in the population, for this continuous model, we assume that all households behave uniformly.)

$$ {\overset{\sim }{S}}_p=\frac{\left|h\right|}{N}{S}_p, $$

$$ {\overset{\sim }{S}}_c=\frac{\left|h\right|}{N}{S}_c, $$

$$ {\overset{\sim }{I}}_p=\frac{\left|h\right|}{N}\ \left(\ {I}_{\mathrm{p}}+{I}_p^{+}\ \right), $$

$$ {\overset{\sim }{I}}_c=\frac{\left|h\right|}{N}\left(\ {I}_c+{I}_c^{+}\ \right), $$

$$ {\overset{\sim }{R}}_p=\frac{\left|h\right|}{N}{R}_p, $$

and

$$ {\overset{\sim }{R}}_c=\frac{\left|h\right|}{N}{R}_c $$

Using these definitions, we define the following initial system of differential equations (which is then modified to include economic constraints). This system highlights the disease dynamics:

$$ \raisebox{1ex}{$d{S}_p$}\!\left/ \!\raisebox{-1ex}{$ dt$}\right.=-{\beta}_P\left({S}_p\left({I}_p+{I}_p^{+}\right)\right)-{\beta}_p\left({S}_p\left({I}_c+{I}_c^{+}\right)\right)-{\iota}_p\left({S}_c{\tilde{I}}_p\right)-{\iota}_p\left({S}_p{\tilde{I}}_c\right), $$

$$ \raisebox{1ex}{$d{S}_c$}\!\left/ \!\raisebox{-1ex}{$ dt$}\right.=-{\beta}_c\left({S}_c\left({I}_p+{I}_p^{+}\right)\right)-{\beta}_c\left({S}_c\left({I}_c+{I}_c^{+}\right)\right)-{\iota}_c\left({S}_c{\tilde{I}}_p\right)-{\iota}_c\left({S}_c{\tilde{I}}_c\right), $$

$$ \raisebox{1ex}{$d{I}_p$}\!\left/ \!\raisebox{-1ex}{$ dt$}\right.=\left({\beta}_p\left({S}_p\left({I}_p+{I}_p^{+}\right)\right)+{\beta}_p\left({S}_p\left({I}_c+{I}_c^{+}\right)\right)+{\iota}_p\left({S}_p{\tilde{I}}_p\right)+{\iota}_p\left({S}_p{\tilde{I}}_c\right)\right)\left(1-{T}_p\right)-{\gamma}_p{I}_p, $$

$$ \raisebox{1ex}{$d{I}_c$}\!\left/ \!\raisebox{-1ex}{$ dt$}\right.=\left({\beta}_c\left({S}_c\left({I}_p+{I}_p^{+}\right)\right)+{\beta}_c\left({S}_c\left({I}_c+{I}_c^{+}\right)\right)+{\iota}_c\left({S}_c{\tilde{I}}_p\right)+{\iota}_c\left({S}_c{\tilde{I}}_c\right)\right)\left(1-{T}_c\right)-{\gamma}_c{I}_c, $$

$$ \raisebox{1ex}{$d{I}_p^{+}$}\!\left/ \!\raisebox{-1ex}{$ dt$}\right.=\left({\beta}_p\left({S}_p\left({I}_p+{I}_p^{+}\right)\right)+{\beta}_p\left({S}_p\left({I}_c+{I}_c^{+}\right)\right)+{\iota}_p\left({S}_p{\tilde{I}}_p\right)+{\iota}_p\left({S}_p{\tilde{I}}_c\right)\right)\left({T}_p\right)-{\gamma}_p^{+}{I}_p^{+}, $$

$$ \raisebox{1ex}{$d{I}_c^{+}$}\!\left/ \!\raisebox{-1ex}{$ dt$}\right.=\left({\beta}_c\left({S}_c\left({I}_p+{I}_p^{+}\right)\right)+{\beta}_c\left({S}_c\left({I}_c+{I}_c^{+}\right)\right)+{\iota}_c\left({S}_c{\tilde{I}}_p\right)+{\iota}_c\left({S}_c{\tilde{I}}_c\right)\right)\left({T}_c\right)-{\gamma}_c^{+}{I}_c^{+}, $$

$$ \raisebox{1ex}{$d{R}_p$}\!\left/ \!\raisebox{-1ex}{$ dt$}\right.={\gamma}_p{I}_p+{\gamma}_p^{+}{I}_p^{+}, $$

and

$$ \raisebox{1ex}{$d{R}_c$}\!\left/ \!\raisebox{-1ex}{$ dt$}\right.={\gamma}_c{I}_c+{\gamma}_c^{+}{I}_c^{+} $$

In each *dS*/*dt* and *dI*/*dt* term, we find the usual *β S I* terms, where in each case, we must account for transmission from all four infective categories. Additional *ι S I* terms account for transmission within households (thus using the $\overset{\sim }{I}$ weighted variables). The *γ I* terms account for recovery in the usual way and are no more complex than the most basic SIR model.

However, these equations have not yet accounted for the economic factors, which change the rates of disease transmission. We reintroduce the variable *M*_*h*_ to indicate the amount of economic resources a household has. To translate this constraint under the averaging effect of the continuous model, *M*_*h*_ represents the economic state of all households, collectively.

For notational simplicity, we define

$$ A=-{\beta}_p\left({S}_p\left({I}_p+{I}_p^{+}\right)\right)-{\beta}_p\left({S}_p\left({I}_c+{I}_c^{+}\right)\right)-{\iota}_p\left({S}_p{\tilde{I}}_p\right)-{\iota}_p\left({S}_p{\tilde{I}}_c\right) $$

$$ B=-{\beta}_c\left({S}_c\left({I}_p+{I}_p^{+}\right)\right)-{\beta}_c\left({S}_c\left({I}_c+{I}_c^{+}\right)\right)-{\iota}_c\left({S}_c{\tilde{I}}_p\right)-{\iota}_c\left({S}_c{\tilde{I}}_c\right) $$

$$ \hat{A}=-{\hat{\beta}}_p\left({S}_p\left({I}_p+{I}_p^{+}\right)\right)-{\hat{\beta}}_p\left({S}_p\left({I}_c+{I}_c^{+}\right)\right)-{\hat{\iota}}_p\left({S}_p{\tilde{I}}_p\right)-{\hat{\iota}}_p\left({S}_p{\tilde{I}}_c\right) $$

$$ \hat{B}=-{\hat{\beta}}_c\left({S}_c\left({I}_p+{I}_p^{+}\right)\right)-{\hat{\beta}}_c\left({S}_c\left({I}_c+{I}_c^{+}\right)\right)-{\hat{\iota}}_c\left({S}_c{\tilde{I}}_p\right)-{\hat{\iota}}_c\left({S}_c{\tilde{I}}_c\right) $$

These $\hat{A}$ and $\hat{B}$ differ from *A* and *B* only by the modified disease transmission rates. We therefore redefine our system of differential equations to include this:

$$ \raisebox{1ex}{$d{S}_p$}\!\left/ \!\raisebox{-1ex}{$ dt$}\right.=\Big\{{\displaystyle \begin{array}{c} if\\ {} if\end{array}}\;{\displaystyle \begin{array}{c}{M}_h>0,\\ {}{M}_h\le 0,\end{array}}\kern1em {\displaystyle \begin{array}{c}A\\ {}\hat{A}\end{array}} $$

$$ \raisebox{1ex}{$d{S}_c$}\!\left/ \!\raisebox{-1ex}{$ dt$}\right.=\Big\{{\displaystyle \begin{array}{c} if\\ {} if\end{array}}\;{\displaystyle \begin{array}{c}{M}_h>0,\\ {}{M}_h\le 0,\end{array}}\kern1em {\displaystyle \begin{array}{c}B\\ {}\hat{B}\end{array}} $$

$$ \raisebox{1ex}{$d{I}_p$}\!\left/ \!\raisebox{-1ex}{$ dt$}\right.=\Big\{{\displaystyle \begin{array}{c} if\\ {} if\end{array}}\;{\displaystyle \begin{array}{c}{M}_h>0,\\ {}{M}_h\le 0,\end{array}}\kern1em {\displaystyle \begin{array}{c}-A\left(1-{T}_p\right)-{\gamma}_p{I}_p\\ {}-\hat{A}-{\gamma}_p{I}_p\end{array}} $$

$$ \raisebox{1ex}{$d{I}_c$}\!\left/ \!\raisebox{-1ex}{$ dt$}\right.=\Big\{{\displaystyle \begin{array}{c} if\\ {} if\end{array}}\;{\displaystyle \begin{array}{c}{M}_h>0,\\ {}{M}_h\le 0,\end{array}}\kern1em {\displaystyle \begin{array}{c}-B\left(1-{T}_c\right)-{\gamma}_c{I}_c\\ {}-\hat{B}-{\gamma}_c{I}_c\end{array}} $$

$$ \raisebox{1ex}{$d{I}_p^{+}$}\!\left/ \!\raisebox{-1ex}{$ dt$}\right.=\Big\{{\displaystyle \begin{array}{c} if\\ {} if\end{array}}\;{\displaystyle \begin{array}{c}{M}_h>0,\\ {}{M}_h\le 0,\end{array}}\kern1em {\displaystyle \begin{array}{c}-A{T}_p-{\gamma}_p^{+}{I}_p^{+}\\ {}-{\gamma}_p{I}_p^{+}\end{array}} $$

$$ \raisebox{1ex}{$d{I}_c^{+}$}\!\left/ \!\raisebox{-1ex}{$ dt$}\right.=\Big\{{\displaystyle \begin{array}{c} if\\ {} if\end{array}}\;{\displaystyle \begin{array}{c}{M}_h>0,\\ {}{M}_h\le 0,\end{array}}\kern1em {\displaystyle \begin{array}{c}-B{T}_c-{\gamma}_c^{+}{I}_c^{+}\\ {}-{\gamma}_c{I}_c^{+}\end{array}} $$

The quantity *M*_*h*_ itself varies over time, based on the number of active resource producers and resource consumers, as follows:

$$ \raisebox{1ex}{$d{M}_h$}\!\left/ \!\raisebox{-1ex}{$ dt$}\right.=\left({P}_p\left({S}_p+{I}_p^{+}+{R}_p\right)-{C}_c\left({S}_c+{I}_c+{I}_c^{+}+{I}_p+{R}_c\right)-{C}_t\left({I}_p^{+}+{I}_c^{+}\right)\right)/N, $$

This formulation permits analysis over a broader set of economic parameters. We vary *C*_*c*_ from 0 to 50 and *C*_*t*_ from 0 to 500. Concretely, with *P*_*p*_ fixed at 50 and a 3:2 consumer to producer ratio, households consume between 0 and 150% of their income daily, and one daily treatment costs between 0 and 5 days’ worth of household income.

To more easily quantify this information, we define CPR to be the consumption-to-production ratio of the average household, and likewise define TCI to be the treatment-cost-index, the number of days’ worth of household income one treatment costs, i.e. $$ TCI=\raisebox{1ex}{${C}_T$}\!\left/ \!\raisebox{-1ex}{$2{P}_p$}\right. $$.

We consider an economy strong when the CPR is low, i.e. where each household consumes significantly less than its daily income, and weak when it is not (in particular, CPR > 1 means households tend to lose money). Using these two relative metrics to understand the economy, we map the entire set of outcomes within the parameter space of *C*_*c*_ and *C*_*t*_.

We allow TCI to range as low as 0 (allowing free treatment) and 5 (an extremely prohibitive cost) in order to analyze a broad set of parameter values. Values of TCI significantly higher than 1 have been observed [33].

We focus much of our attention in each instance to lines of demographically proportionate treatment, by which we mean gradations in the *T*_*c*_ − *T*_*p*_-plane wherein equal numbers of treatments are administered, allocated differently between producers and consumers. The slope of these lines reflects the 3:2 ratio of consumers to producers. These are indicated in all graphs of the *T*_*c*_ − *T*_*p*_-plane by white lines. We discuss a number of comparable scenarios in which the economic parameters are fixed and we contrast two (or more) points in the *T*_*c*_ − *T*_*p*_-plane and the outcomes therein. The comparable points are along lines of demographically proportionate treatment – where the combined total of individuals treated would be equal, assuming otherwise proportionate incidence of the disease – the neutral assumption to make in order to test hypotheses related to disproportionate disease burden, and a reflection of treatment strategies that are predetermined, not dynamic.

It is important to note that the purpose of this study is to understand how the effect of household-level resource limitations interact with treatment strategies that are devised a priori. We are considering treating a fixed portion of each category of the population, assuming with no future knowledge of the outbreak and no means for more complex adaptive or otherwise time-varying treatment strategy. In resource-limited environments, it may not be possible to implement a strategy besides a static, predetermined allocation of a certain treatment rate for each of the two categories, treating a given fraction of producers and consumers presenting with the disease.

To demonstrate the impact of household-level resource limitation, we explore the parameter space of this model, varying the economic parameters (CPR and TCI) and the treatment coverages (*T*_*p*_ and *T*_*c*_). We observe scenarios in which, for a fixed CPR and TCI, varying *T*_*p*_ and *T*_*c*_ in a way that allocates preferential treatment for consumers (while treating equivalently fewer consumers) *increases* the disease burden within that vulnerable group.

## Results

**Fig. 1:**
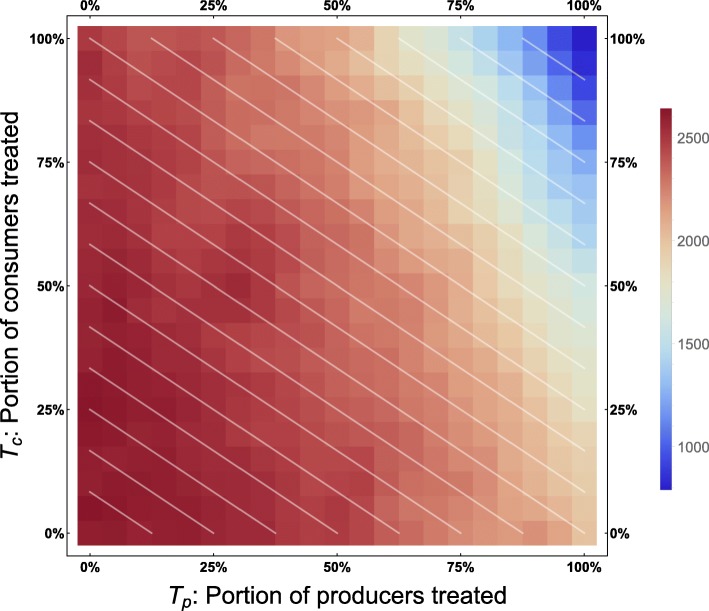
Consumer disease burden vs. treatment protocol. Discrete model, with economic parameters CPR = 0.6, TCI = 1, and all other parameters as in Table 1. Lines of demographically proportionate treatment in white. Measured as total individuals infected

In the discrete model, Monte Carlo simulations reveal preferentially treating consumers, given a fixed number of total individuals treated, generally leads to a *higher* incidence of consumer infection. This phenomenon appears throughout the parameter space (Fig. 1).

**Fig. 2:**
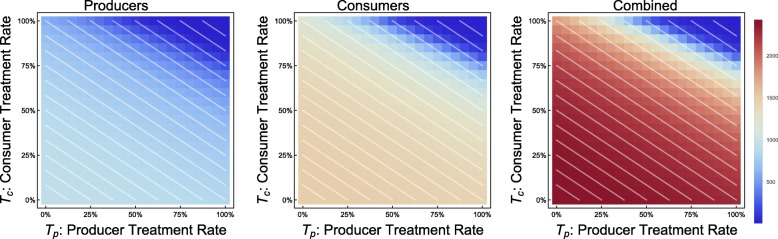
Incidence of infection vs. treatment protocol. Continuous model with economic parameters CPR = 0.6, TCI = 0.5

This phenomenon is also supported by results from the continuous model. In cases in which the economy is strong or treatment is inexpensive, we see the type of behavior we would naïvely expect: along lines of demographically proportionate treatment in the *T*_*p*_ − *T*_*c*_ plane, the incidence of infection in producer and consumer populations remains relatively constant. As treatment increases for either group, a steady improvement in the outcomes is visible (e.g. CPR = 0.6, TCI = 0.5, see Fig. 2).

**Fig. 3:**
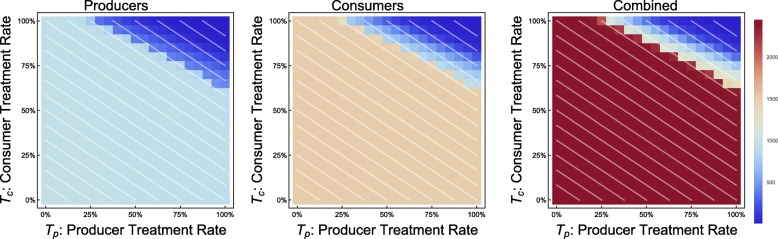
Incidence of infection vs. treatment protocol. Continuous model with economic parameters CPR = 0.9, TCI = 5

In the case of an extremely weak economy, we see outcomes that are similarly naïve, in that there is no discernable difference between incidences of infection along lines of demographically proportionate treatment. The significant difference between this and a stronger economic scenario is a sharp rise in disease incidence with only minor decreases in the treatment coverage (e.g. CPR = 0.9, TCI = 5, see Fig. 3).

**Fig. 4:**
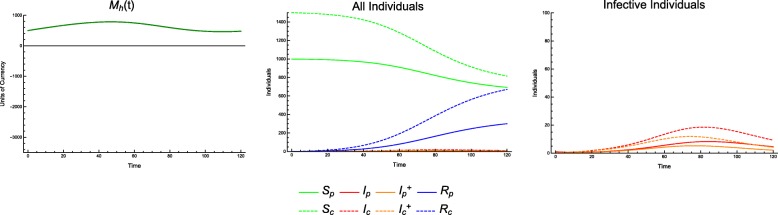
Incidence of infection over time. Continuous model with economic parameters CPR = 0.9, TCI = 5 and treatment coverage *T*_*p*_ = 0.8, *T*_*c*_ = 0.8

**Fig. 5:**
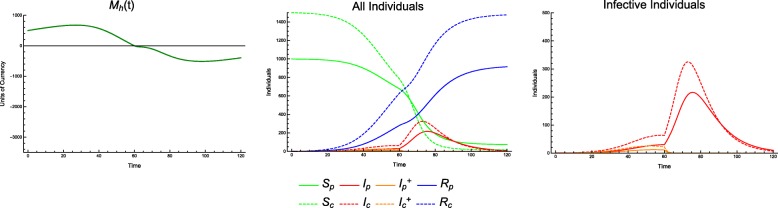
Incidence of infection over time. Continuous model with economic parameters CPR = 0.9, TCI = 5 and treatment coverage *T*_*p*_ = 0.7, *T*_*c*_ = 0.7

This sharp threshold in outcomes for a weak economy highlights the role of the economy in epidemiological outcomes. With *T*_*p*_ = 0.8 and *T*_*c*_ = 0.8 (Fig. 4), we see a lower incidence of disease and an economy that *almost* goes bankrupt (bankrupt meaning *M*_*h*_ ≤ 0). With *T*_*p*_ = 0.7 and *T*_*c*_ = 0.7 (Fig. 5), we see a slightly larger incidence of disease before the economy goes bankrupt, but after it does so, the incidence rises sharply.

Recall that each treatment scenario does not imply treatment of all cases. For example, *T*_*p*_ = 0.75 means treatment of 75% of cases in producers so long as there are sufficient economic resources to provide treatment at the onset of each infection. Once these resources are depleted, additional individuals are not treated. This depletion can result in secondary jump or other bimodal behavior in the infective population. It is thus possible for alternative strategies to allow for better allocation of economic resources, provide more effective control even with *fewer* cases specified for treatment.

**Fig. 6:**
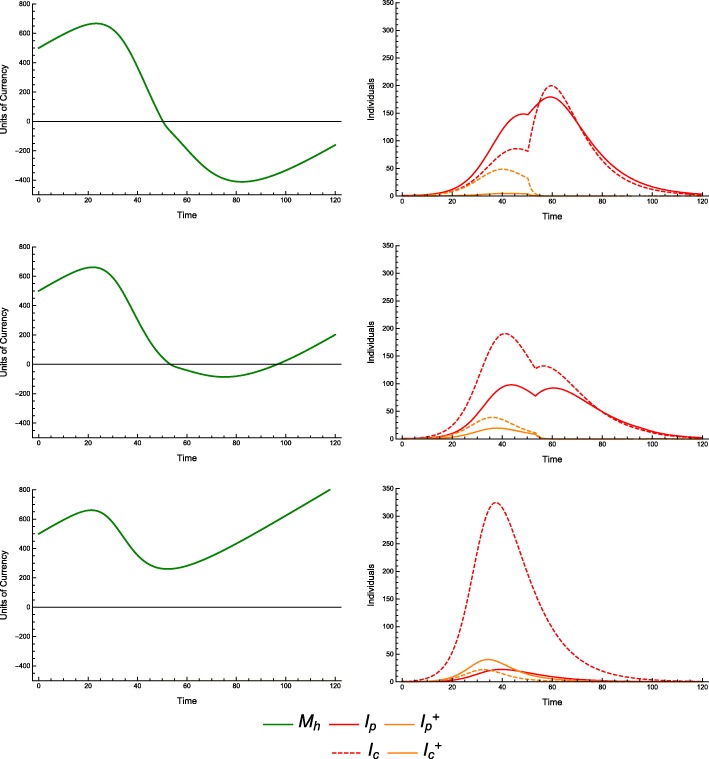
Incidence of infection over time. Continuous model, with economic parameters CPR = 0.9, TCI = 2.5; treatment coverage: *T*_*p*_ = 0.15, *T*_*c*_ = 0.75 (top), *T*_*p*_ = 0.51, *T*_*c*_ = 0.51 (middle), *T*_*p*_ = 0.9, *T*_*c*_ = 0.25 (bottom); and all other parameters as in Table 1. While *T*_*c*_ = 0.25 shows a higher peak incidence in consumers, as one might expect, *T*_*c*_ = 0.75 has a lengthier outbreak and higher overall incidence in consumers

For example, consider a scenario with CPR = 0.9 and TCI = 2.5. We can treat the same number of individuals in three different ratios (producers to consumers), either favoring consumers, favoring producers, or treating equal numbers of each (Fig. 6).

Using the case of equal treatment (*T*_*p*_ = 0.51 and *T*_*c*_ = 0.51) as a baseline (1450 consumers infected), we find that by favoring consumers (*T*_*p*_ = 0.15 and *T*_*c*_ = 0.75), the incidence of infection in consumers *increases* by 1.6% (1473 infected). Conversely, favoring producers (*T*_*p*_ = 0.9 and *T*_*c*_ = 0.25), the consumer incidence *decreases* by 4.6% (1383 infected).

Note that we are comparing outcomes along lines of demographically proportionate treatment (see detailed explanation in Methods above). This distinction allows us to consider the question of whom to treat (i.e., which proportion of each class of individuals to treat) given a fixed number of treatments, in order to have a particular outcome. We are not considering cases in which we treat *equal* numbers of producers and *more* consumers, which in all cases lead to better outcomes for both groups. We are considering cases in which, in order to treat more consumers, we treat *fewer* producers. We have found that for certain economic parameters, this is *worse* for consumers.

**Fig. 7:**
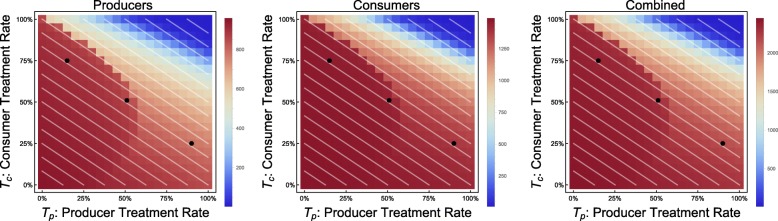
Incidence of infection vs. treatment protocol. Continuous model, with economic parameters CPR = 0.9, TCI = 2.5, and all other parameters as in Table 1. Treatment coverages shown in Fig. 6 marked with black dots

**Fig. 8:**
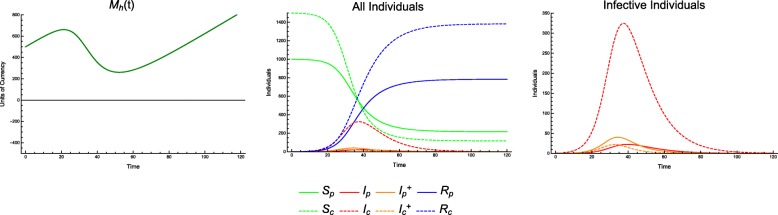
Incidence of infection over time. Continuous model with economic parameters *C*_*c*_ = 30, *C*_*t*_ = 250 and treatment coverage *T*_*p*_ = 0.15, *T*_*c*_ = 0.75

**Fig. 9:**
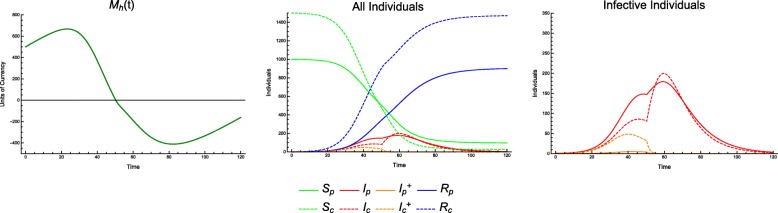
Incidence of infection over time. Continuous model with economic parameters *C*_*c*_ = 30, *C*_*t*_ = 250 and treatment coverage *T*_*p*_ = 0.51, *T*_*c*_ = 0.51

**Fig. 10:**
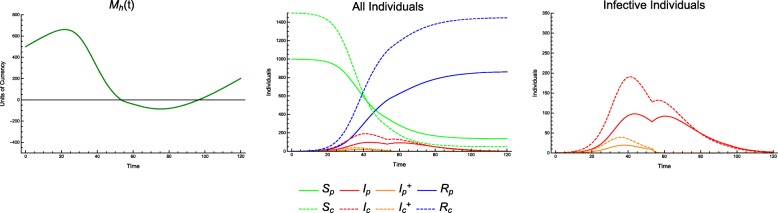
Incidence of Infection over Time. Continuous model with economic parameters *C*_*c*_ = 30, *C*_*t*_ = 250 and treatment coverage *T*_*p*_ = 0.9, *T*_*c*_ = 0.25

This phenomenon is easily explained. Although equal numbers of individuals are treated, by treating more producers, the economy continues to be healthier, with *M*_*h*_(*t*) > 0. By treating more consumers, we find that the economy can temporarily or permanently run out of resources (i.e. *M*_*h*_(*t*) < 0). Treating even more producers can prevent or shorten this bankruptcy, resulting in even lower incidence among consumers. This is a case in which we could observe in the *T*_*p*_ − *T*_*c*_ plane where outcomes are no longer equivalent along lines of demographically proportionate treatment, as shown in Fig. 7. These scenarios are also shown in greater detail in Figs. 8, 9 and 10.

Additionally, we can observe that the epidemiological dynamics are affected significantly by the transition from *M*_*h*_(*t*) > 0 to *M*_*h*_(*t*) < 0. Treating more consumers makes this phenomenon worse, resulting in longer periods in which the economic resources available for treatment are exhausted, during which there will be an increase in incidence of disease (both overall, and specifically in consumers). The transition to an economic state of bankruptcy induces a second wave of the outbreak, and it is clear (a priori, or from the data) that the length of this secondary outbreak is worse than the first, since *M*_*h*_(*t*) < 0 increases the disease transmission and decreases the recovery.

By reframing this model as a system of differential equations, key quantities (in particular, *M*_*h*_) are no longer distributed across households – they are simply averaged. Instead of seeing this phenomenon steadily (Fig. 1), we see sharper thresholds with more pronounced jumps (see Figs. 3 and 7 – particularly Fig. 3 contrasted with Fig. 2), but only along lines of demographically proportionate treatment near the center of the graph, rather than throughout the entire parameter space (as in the discrete case).

We note that these jumps (non-smooth points) at the start of a secondary outbreak are, in some sense, artifacts of the differential equations model, wherein households experience similar conditions at the same points in time. In the agent-based simulation, we have observed the same phenomenon, but with the heterogeneity of a discrete, stochastic model, the two outbreaks are not separated by a specific non-smooth point at some point where a threshold is crossed.

**Fig. 11:**
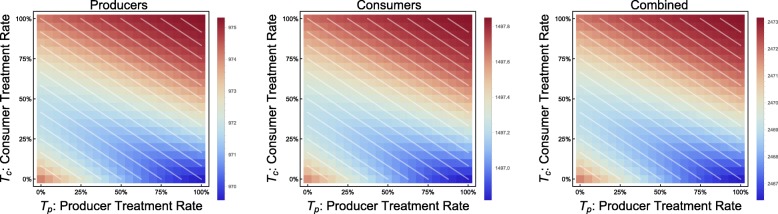
Incidence of infection vs. treatment protocol. Continuous model with economic parameters CPR = 1.2, TCI = 1.5

Another counter-intuitive phenomenon appears in the continuous model at a second-order level. When no other phenomena occur in the *T*_*p*_ − *T*_*c*_ plane, we find very little variation in incidence (e.g. CPR = 1.2, TCI = 1.5, see Fig. 11).

However, we see instead second-order behavior that shows it may be possible to allocate too many economic resources on treatment (for either producers or consumers). We can observe that in certain cases, higher treatment coverage result in the depletion of economic resources early, resulting in an effective treatment coverage much lower, while slightly lower coverage allows the economy to sustain itself for *slightly* longer. (While this gain is truly very slight within the scope of our current model, more detailed economic models may provide greater insight into critical cases; the marginality of this benefit is what makes this effect a very small, second-order phenomenon.)

We have characterized three distinct phenomena that may occur in the *T*_*p*_ − *T*_*c*_, depending on the values of *C*_*c*_ and *C*_*t*_ (with other parameters fixed). We name them as follows:

The *weak economy* phenomenon, where there is a sharp decrease in the incidence of disease when treatment level reaches a certain threshold (treatment of fixed number of individuals, regardless of whether they are consumers or producers), as demonstrated in the example in Fig. 3;

The *threshold* phenomenon, where shifting from consumer-heavy treatment strategies towards producer-heavy treatment strategies produces a paradoxical benefit for consumers, even moving along lines of demographically proportionate treatment, as in the example in Fig. 7;

The *overspending* phenomenon, the second-order phenomenon demonstrated in Fig. 11.

**Fig. 12:**
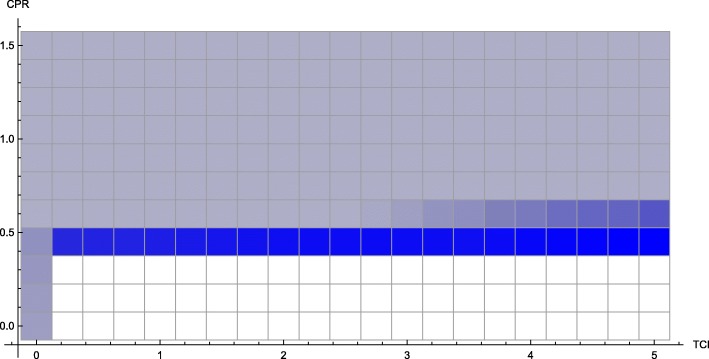
Occurrence of “threshold” phenomenon in the *C*_*c*_ − *C*_*t*_ plane. Continuous model with all parameters as in Table 1. Brighter blue represents a greater magnitude of the phenomenon, measured in the greatest relative drop in disease burden for consumers along a line of demographically proportionate treatment

**Fig. 13:**
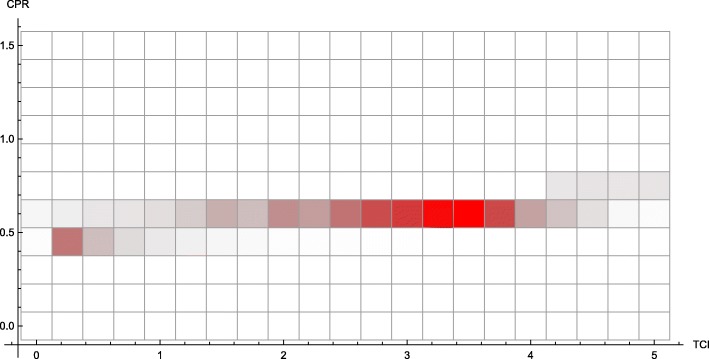
Occurrence of “weak economy” phenomenon in *C*_*t*_ − *C*_*c*_ plane

**Fig. 14:**
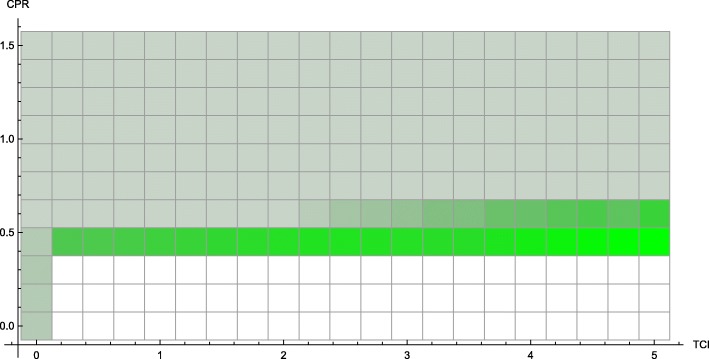
Occurrence of “overspending” phenomenon in *C*_*t*_ − *C*_*c*_ plane

We can quantify the occurrence of these phenomena in the *C*_*c*_ − *C*_*t*_ plane. We show the most remarkable, the threshold phenomenon, in Fig. 12. We can observe that these phenomena all occur most prominently in cases in which CPR is 0.45 to 0.75, a range in which the cost of consumers is significant, but not too close to the total household income. The other two phenomena are also shown individually as Figs. 13 and 14, respectively.

## Discussion

The majority of epidemiological studies exploring the optimal patterns of limited resource allocation to achieve maximal epidemic control have assumed population level economic or biomedical limitations. While these assumptions work well for the developed world, in the developing world there are many different scenarios of economically determined disease susceptibility. As we have begun to explore, these scenarios can depend upon the personal, household maintenance of health, including minimal, ongoing, adequate nutrition, but also clearly need to incorporate the underlying demography, relative levels of financial productivity, treatment costs, and socioeconomic distribution of the population to be protected. Critically, however, exactly such scenarios are those in which subsidized availability of medical interventions and outcome-based targeted public health campaigns are most frequently employed.

For tropical diseases in areas where household treatment strategies are limited by the day-to-day income of working adults, we have demonstrated that it is important to consider the economic productivity as a crucial contribution to the health of other members of the household. Diseases like malaria, Chagas disease, and leptospirosis, whether endemic or emergent, in resource-limited communities, should require careful analysis before making decisions regarding targeted treatment strategies, particularly when triggered by natural disasters, which can themselves temporarily depress the economic productivity and elasticity of a vulnerable community.

Naturally, the models here presented apply directly in only a very explicit set of circumstances: illness must be sufficiently debilitating as to prevent someone from going to work, and financial support must be able to provide sufficient direct impact to health status as to affect susceptibility to infectious disease, and treatment must speed recovery without increasing the ability to transmit the disease in the meanwhile by enabling nominal social interactions. While these requirements are far from rare in the developing world, analysis of the specifics will be critical to any recommendation as to effective targeted treatment strategy. These models have demonstrated the need for these specific investigations, showing how individual-level economic constraints and disease etiology can invalidate the accuracy of recommendations from models that fail to incorporate these concerns. Based on these results, it is clear that targeted policies, meant to protect the most vulnerable members of a population, may inadvertently backfire if the broader economic impacts of intervention are not also considered.

## Conclusions

Using both agent-based simulation and continuous differential equations, we explore the implications of expanding traditional epidemiological models to account for individual- and family-level economic factors: the production and consumption of household resources. In this resources-limited model, treatment is allocated only when a household can afford it. We answer a key question: which individuals in each household to treat, producers or consumers. Although the model assumes certain economic circumstances and represents a small, well-mixed population, it exhibits a phenomenon worthy of study and indicating caution is required.

We analyze the relationship between the total cases of consumer illness, as a measure of adverse outcomes in children, against three parameters: the net-income of each household, the cost of treatment, and the proportion treated from each group (producers and consumers). We examine household-scale economies in which a family does not have a high net-income relative to the cost of treatment. The most effective strategy for minimizing the number of consumers, the at-risk group, to contract the disease may be to *focus treatment on the other group*, the producers. This counter-intuitive conclusion is demonstrated by examining how the economy impacts the overall dynamics of the disease outbreak. This suggests further study, in more diverse systems representing less homogeneous systems or more specific populations, is necessary and that public health policy should be informed by this ongoing study.

We also suggest that increasing economic inequality, on the global scale, has created or entrenched low-income communities like the ones we describe all over the world, living day-to-day without monetary resources to weather a health crisis. For this reason, there is great importance in research that models such communities and accounts for nuances of health policy for these peoples and regions, particularly when a more refined or nuanced approach might run counter to naïve intuition or best practices in other environments.

## Supplementary information

**Additional file 1.** Supplemental File 1: Model implementations in the Wolfram language.

## Data Availability

The computer code used to implement these methods, as well as the datasets generated by Monte Carlo simulation, are available from the corresponding author on reasonable request.

## Abbreviations

CPR: Consumption-to-Production Ratio, ratio of the average household’s resource
SIR: Susceptible-Infective-Recovered, standard compartmental model for epidemics
TCI: Treatment Cost Index, the number of days’ worth of household income one treatment costs
